# HSP27 inhibitor attenuates radiation-induced pulmonary inflammation

**DOI:** 10.1038/s41598-018-22635-9

**Published:** 2018-03-08

**Authors:** Jee-Youn Kim, Yong-Min An, Byeong Rok Yoo, Jin-Mo Kim, Song Yee Han, Younghwa Na, Yun-Sil Lee, Jaeho Cho

**Affiliations:** 1 Department of Radiation Oncology, Yonsei University College of Medicine, Seoul, Republic of Korea; 2 College of Pharmacy, CHA University, Pocheon, 487-010 Republic of Korea; 3 College of Pharmacy and Division of Life and Pharmaceutical Science, Ewha Womans University, Seoul, Republic of Korea

## Abstract

Radiation therapy has been used to treat over 70% of thoracic cancer; however, the method usually causes radiation pneumonitis. In the current study, we investigated the radioprotective effects of HSP27 inhibitor (J2) on radiation-induced lung inflammation in comparison to amifostine. In gross and histological findings, J2 treatment significantly inhibited immune cell infiltration in lung tissue, revealing anti-inflammatory potential of J2. Normal lung volume, evaluated by micro-CT analysis, in J2-treated mice was higher compared to that in irradiated mice. J2-treated mice reversed radiation-induced respiratory distress. However, amifostine did not show significant radioprotective effects in comparison to that of J2. In HSP27 transgenic mice, we observed increased immune cells recruitment and decreased volume of normal lung compared to wild type mice. Increased ROS production and oxidative stress after IR were down-regulated by J2 treatment, demonstrating antioxidant property of J2. The entire data of this study collectively showed that J2 may be an effective therapeutic agent for radiation-induced lung injury.

## Introduction

Radiation therapy is widely used to treat thoracic cancers^1^. However, lung tissues are relatively sensitive to radiation^2^. Consequently, radiation-induced lung injury (RILI) is classified as early-phase pneumonitis, and about 13–37% of patients receiving radiotherapy for lung cancer were susceptible to developing radiation pneumonitis^3^ or late-phase fibrosis. Pneumonitis is characterized by appearance of alveolar edema, infiltration of inflammatory cells, and aggregation of hyaline products^4^. Infiltrated inflammatory cells are activated to release various cytokines, such transforming growth factor (TGF)-β and interleukin (IL)-1b^5^. Excessive inflammation accelerates collagen and extracellular matrix formation, resulting in tissue fibrosis^5,6^.

Pneumonitis and fibrosis are predominant consequences of radiation exposure^7^, and numerous studies have indicated that inflammation plays a crucial role in the development of fibrosis. Therefore, the pathology of pneumonitis is closely associated with fibrosis. Recently, it has been reported that increase of heat shock protein 27 (HSPB1, HSP27 in humans and HSP25 in mouse) is associated with fibrosis progress. HSP27, which protects against cellular stress, is overexpressed in various cancers. Depletion of HSP27 in cancer model induces tumor regression^8^. Overexpression of HSP27 is known to increases drug resistance in several cancer cells^9^, which means HSP27 might be an attractive target for cancer therapy. Nevertheless, only two HSP27 inhibitors are under clinical trial. One was the antisense oligonucleotide OGX-427, and the other was RP101. There were no small molecules developed as HSP27 inhibitors in cancer treatment except RP101. We have previously demonstrated that synthetic compounds zerumbone and SW-15 could induce abnormal cross-linking of the HSP27 protein. Altered crosslinking of HSP27 modifies normal HSP27 dimerization resulting in functional inhibition of HSP27, thereby sensitizing tumors to conventional radiation and chemotherapies^10,11^. As an continuous study to further optimize HSP27 inhibitors through abnormal dimerization of HSP27, we have additionally designed and synthesized a small number of chrome-4-one derivatives and found J2 (Supplementary Fig. S1A), which effectively created abnormal HSP27 cross-linking and showed better therapeutic efficiency in radiation or chemotherapeutic treatment.^12,13^.

Stereotactic body radiotherapy (SBRT) is a recently developed technique that delivers high doses of ablative radiation to tumors in a single fraction, with greater accuracy than conventional fractionated radiotherapy (CFRT). It has become the standard radiotherapy method for early-stage lung cancer^14–16^ However, there has been a lack of relevant mouse models for evaluating the effects of ablative radiation doses *in vivo*. In our previous study, we established an experimental radiation-induced lung inflammation mouse model using an image-guided animal irradiation method similar to SBRT^17^ to deliver a single dose of 75 Gy to the left lung of mice. The mice exhibited radiation pneumonitis at two weeks post-irradiation. In these models, we found that HSP27 expression increased significantly. Therefore, we hypothesized that HSP27 might be involved in radiation-induced lung inflammation, which could be overcome through inhibition of HSP27.

Amifostine, a synthetic sulfhydryl compound, is the first and only drug approved by the U.S. Food and Drug Administration as a radioprotective drug^18^. However, use of amifostine has been limited to head and neck cancer patients, owing to its side effects such as diarrhea, hypotension, hypocalcemia, and neurotoxicity. In this study, we focused our investigation on the role and mechanism of J2 in radiation-induced pneumonitis and lung function in mice model, in comparison to that of amifostine.

## Results

### Effect of J2 on radiation-induced morphological and histological changes in lung tissue

**Figure 1:**
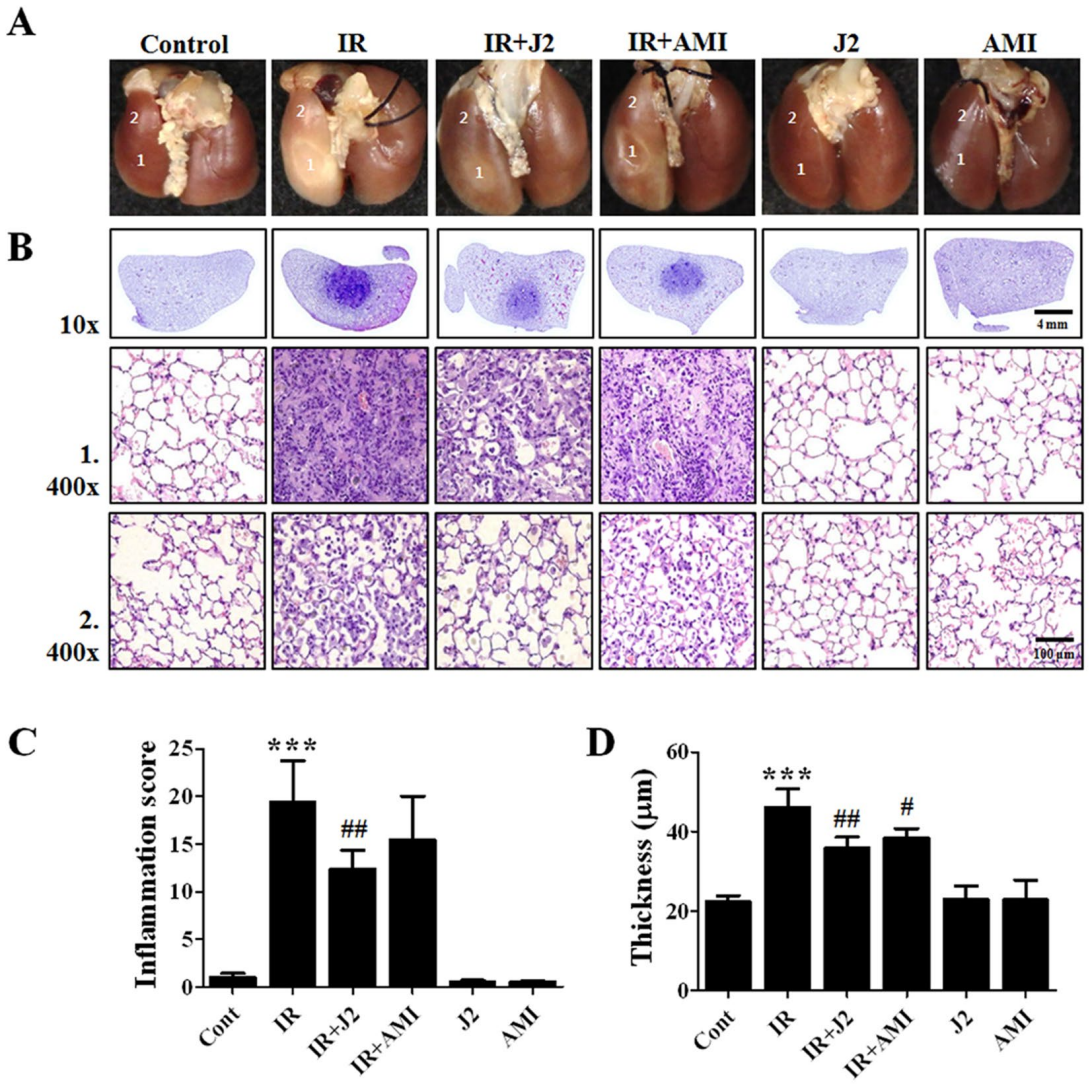
Effect of HSP27 inhibitor on gross morphology and histopathological analysis. (**A**) Representative gross finding. Mice were sacrificed at two weeks after irradiation. Four-percent paraformaldehyde was instilled via trachea, and lungs were immersed in fixation solution and photographed after complete fixation. (**B**) Haematoxylin and eosin-stained lung sections. Numbers 1 and 2 indicate areas of radiation-induced injury and spread of inflammation toward outside of radiation-induced injury. Magnification, 10×, 400×. (**C**) Quantification of inflammatory score. (**D**) Thickness of bronchiolar epithelium. Results are expressed as mean ± standard error. ***P < 0.001, versus control; ^#^P < 0.05 and ^##^P < 0.01 versus IR. Control, untreated; IR, 75 Gy irradiation (IR); IR + J2, irradiation + 15 mg/kg J2; IR + AMI, irradiation + 100 mg/kg amifostine; J2, 15 mg/kg J2 only and AMI, 100 mg/kg amifostine only.

To evaluate the effect of radiation on lung morphology, we observed morphological changes of lung tissue samples. In comparison to normal control, irradiated areas in the left lung clearly exhibited local injury. In contrast to the brown color of lungs in control mice, lungs of irradiated mice exhibited a definite, white, ring-like boundary (Fig. 1A, marked as 1) with white-colored adjacent areas (Fig. 1A, marked as 2), indicating lung inflammation. Compared with normal control, in which alveolar septum was clear without inflammatory cell infiltration, accumulation of abundant neutrophils, mononuclear cells in alveoli, destruction of alveolar septa, and intra-alveolar hyaline membrane formation in IR group were significantly higher (Fig. 1 B,C). The thickening of bronchiolar epithelium, which may be caused by inflammation^19^, was significantly increased in IR group supporting hyperplasia of the bronchiolar epithelium (Fig. 1D). However, we could not observe any histological abnormalities in the right lung compared to those observed in the left lung (Supplementary Fig. S2). Overall, the histological study revealed a radiation-induced inflammation confined to the left lung in our mice model. J2-treated mice (IR + J2) exhibited reduced amount of gross and histological changes compared to IR. Interestingly, these protective effects by J2 were higher than those of amifostine.

### Micro-computed tomography findings

**Figure 2:**
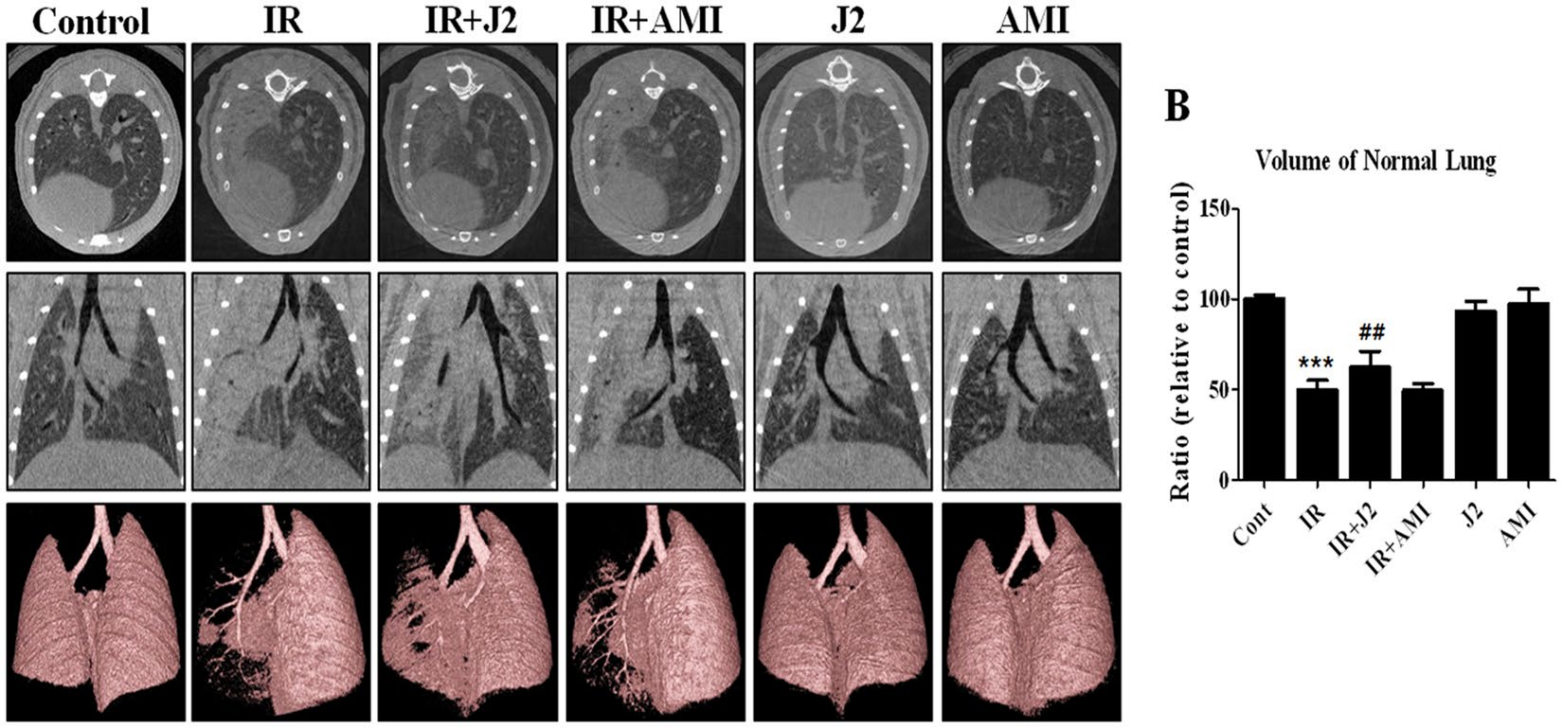
Micro-computed tomography (CT) findings. Representative micro-CT images of lungs of irradiated and control mice. (**A**) Horizontal (top row), trans-axial (middle row), and 3D micro-CT (bottom row) images acquired at two weeks after irradiation. (**B**) Quantification of volume of normal left lung was presented as mean ± standard error. **P < 0.01 versus control; ^##^P < 0.01 versus IR. Control, untreated; IR, 75 Gy irradiation (IR); IR + J2, irradiation + 15 mg/kg J2; IR + AMI, irradiation + 100 mg/kg amifostine; J2, 15 mg/kg J2 only and AMI, 100 mg/kg amifostine only.

Computed tomography (CT) images of lung may use to predict of pneumonitis. Micro-CT is comparable with clinical CT in human^20^, which has been increasingly used in pulmonary research for investigating inflammation in mice models. Representative micro-CT images visualizing progressive anatomical changes in lungs of both irradiated and control mice are shown in Fig. 2A. In inflammatory areas, accumulation of inflammatory exudate (consolidation) was present in the alveoli, preventing air access. These regions appeared as gray areas in micro-CT images. Two weeks after irradiation, pulmonary consolidation was observed throughout the left lungs of irradiated mice. In contrast, J2-treated mice exhibited fewer areas of consolidation. The 3D images provide a more intuitive indication of the lung damage than do the 2D images and support the similar findings.

As shown in Fig. 2B, normal volume of lung in IR group was lower compared to that in control mice. However, in IR + J2 group, the volume appears to have significantly recovered compared to IR. Moreover, in consistence with the gross results, J2 was more effective than amifostine in the volume of normal lungs.

### Effects of J2 on functional parameters of lung

**Figure 3:**
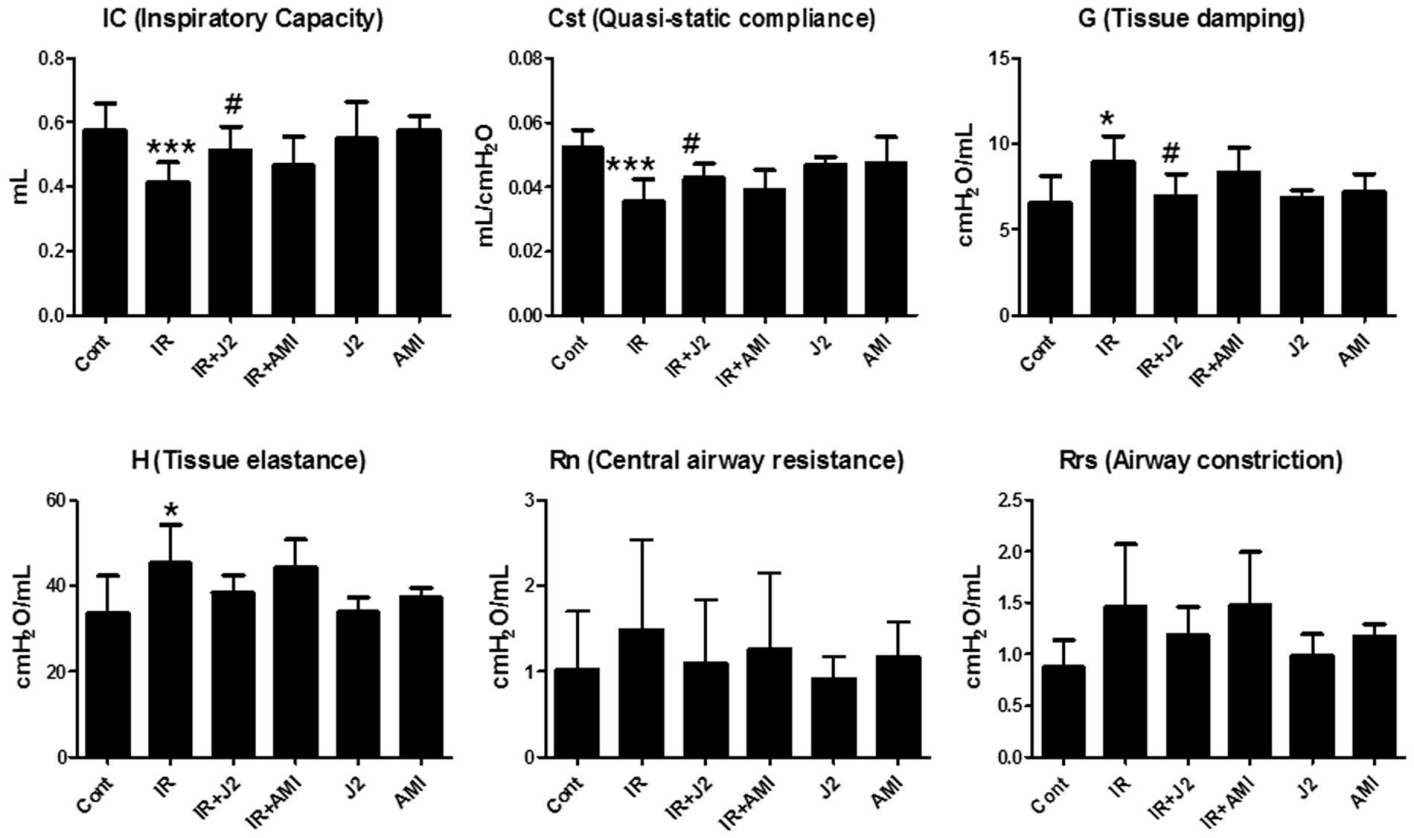
Effect of HSP27 inhibitor on functional evaluation of lungs in irradiated mice. Functional measurements of mouse lungs were collected with Flexivent system at two weeks after irradiation (*P < 0.05 and ***P < 0.001 versus control; ^#^*P* < 0.05 vs. IR). (**A**) IC; inspiratory capacity (**B**) Cst; quasi-static compliance (**C**) G; tissue damping (**D**) H; tissue elastance (**E**) Rn; central airway resistance (F) Rrs; airwayconstrion. Control, untreated; IR, 75 Gy irradiation (IR); IR + J2, irradiation + 15 mg/kg J2; IR + AMI, irradiation + 100 mg/kg amifostine; J2, 15 mg/kg J2 only and AMI, 100 mg/kg amifostine only.

Radiation-induced changes of lung function were evaluated by Flexivent system. Six functional lung parameters were evaluated in this study, and they have been listed in Supplementary Table S1. Among these six parameters, significant differences were observed in inspiratory capacity (IC), quasi-static compliance (Cst), tissue damping (G), and tissue elastance (H) of lungs between IR group and control mice. IC and Cst of control group (0.58 ± 0.031 mL and 0.05 ± 0.002 mL/cmH_2_O, respectively) were significantly higher compared to those of IR group (0.41 ± 0.022 mL [*P* < 0.001] and 0.035 ± 0.003 mL/cmH_2_O [*P* < 0.001], respectively). Values of G and H in control group (6.57 ± 0.596 cmH_2_O/mL and 33.59 ± 3.315 cmH_2_O/mL, respectively) were lower compared to those in IR group (8.95 ± 0.53 cmH_2_O/mL [*P* < 0.05] and 45.21 ± 3.147 cmH_2_O/mL [*P* < 0.05], respectively) (Fig. 3). These results reflect the respiratory distress induced by irradiation in IR group. However, radiation-induced respiratory distress in IR + J2 group appears to have been significantly reversed in IC, Cst, and G parameters, while IR + AMI group failed to reverse radiation-induced respiratory distress, which indicates that J2 has a protective effect on radiation-induced lung injury and is more effective than amifostine.

### Effects of J2 on inflammation-related gene expressions in lung tissue

**Figure 4:**
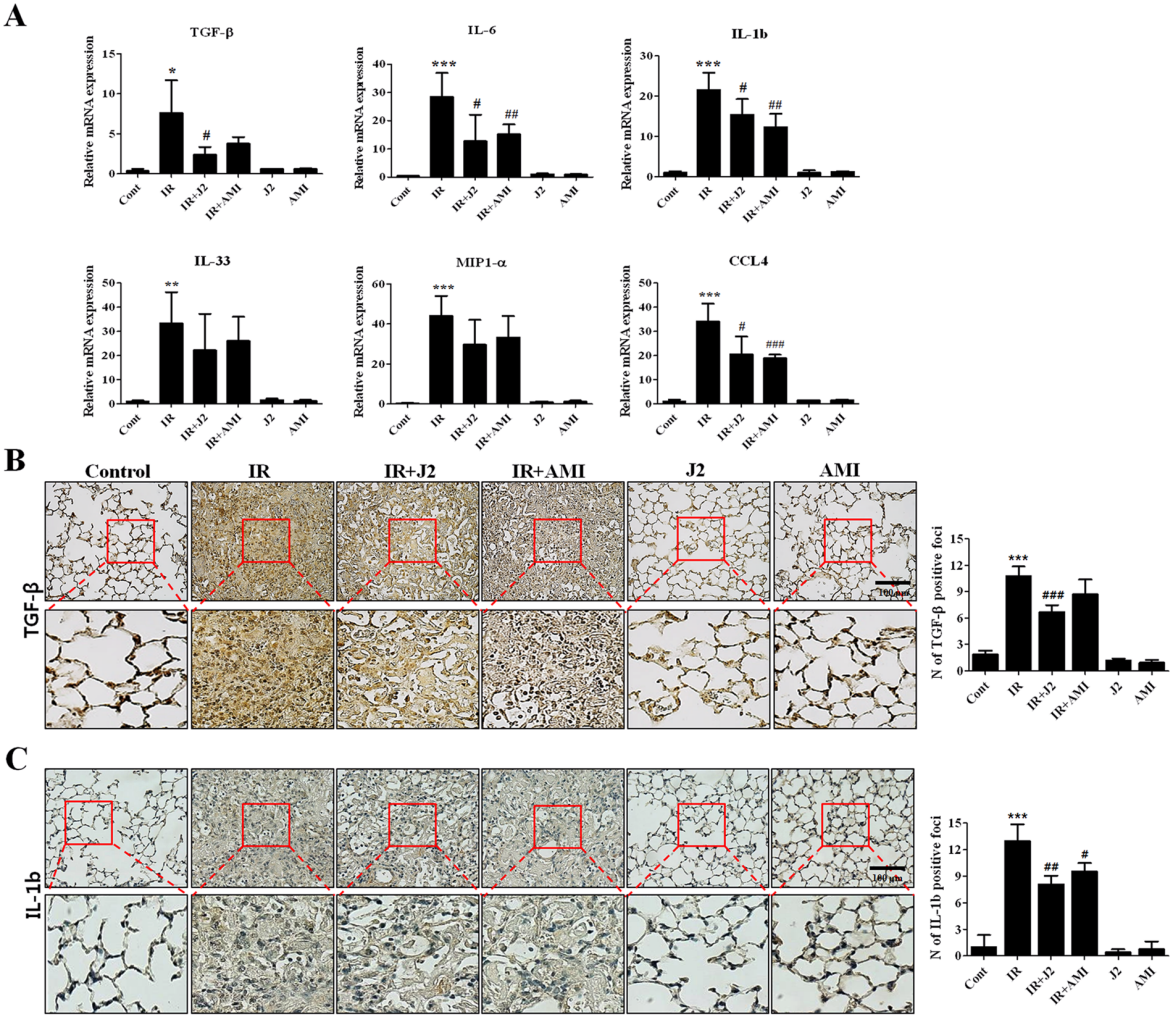
HSP27 inhibitor decreased the expression of inflammation-related molecules. (**A**) cDNA was synthesized from the total RNAs extracted from mouse lungs exposed to X-rays and subjected to quantitative real-time PCR analysis for inflammation-related molecules. (**B**) Immunohistostaining of TGF-β (**B**) and IL-1b (**C**) using mice lung tissues. Quantification of stained tissue was presented as mean ± standard error. *P < 0.05, **P < 0.01 and ***P < 0.001 versus control; ^#^*P* < 0.05, ^##^*P* < 0.01 and ^###^*P* < 0.001 vs. IR. Control, untreated; IR, 75 Gy irradiation (IR); IR + J2, irradiation + 15 mg/kg J2; IR + AMI, irradiation + 100 mg/kg amifostine; J2, 15 mg/kg J2 only and AMI, 100 mg/kg amifostine only.

Next, we evaluated mRNA expressions of cytokines (TGF-β, IL-6, IL-1b and IL-33 and) and chemokines (MIP1-a and CCL4). As shown in Fig. 4A, mRNA levels of cytokines and chemokines were markedly elevated in IR group. Treatment with J2 reduced mRNA levels of these genes, compared to IR or IR + AMI group (Fig. 4A). Immunohistochemical findings revealed that both TGF-β and IL-1b levels increased by IR in lung tissues were decreased in IR + J2 group, compared to that of IR or IR + AMI group (Fig. 4B).

### J2 reduced radiation-induced oxidative stress

**Figure 5:**
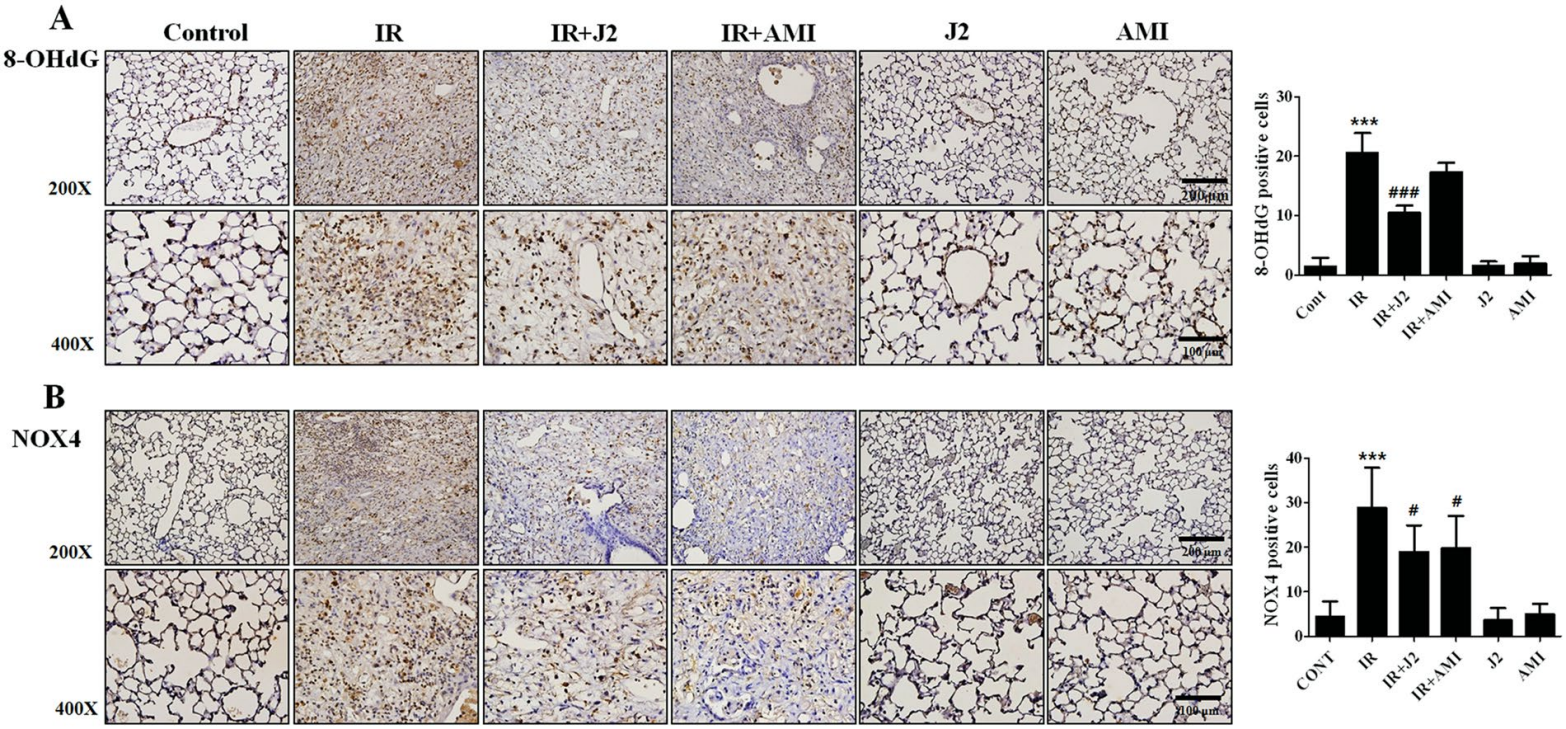
Effects of HSP27 inhibitor on oxidative stress in irradiated mice lungs. Oxidative stress for 8-OHdG (**A**) and NOX4 (**B**) were evaluated in mice lung tissues. Quantification of stained tissue was presented as mean ± standard error. Magnification, 200×, 400×. ***P < 0.001 versus control; ^#^*P* < 0.05 and ^###^*P* < 0.001 vs. IR. Control, untreated; IR, 75 Gy irradiation (IR); IR + J2, irradiation + 15 mg/kg J2; IR + AMI, irradiation + 100 mg/kg amifostine; J2, 15 mg/kg J2 only and AMI, 100 mg/kg amifostine only.

Oxidative stress is a major driving mechanism of lung tissue damage after radiation^21^. Therefore, we evaluated oxidative stress using immunohistochemistry for 8-OHdG and NOX4 in irradiated lung. As shown in Fig. 5, 8-OHdG and NOX4 level were increased in IR group, which was significantly decreased by treatment with J2. Amifostine has been known to have an antioxidant ability against radiation^22^. However, as shown in Fig. 5 A,B, the levels of oxidative stresses were higher in IR + AMI group than in IR + J2 group. These results suggest that J2 might have antioxidant activity. Therefore, we confirmed the antioxidant activity of J2 through DPPH assay. DPPH radical scavenging activity was increased in J2 treatment with dose-dependent manner (Supplementary Fig. S3), suggesting that J2 had antioxidant activity.

Our data also suggested that J2 is likely to have stronger antioxidant property than amifostine.

### J2 reduced ROS generation and inflammasome activation

**Figure 6:**
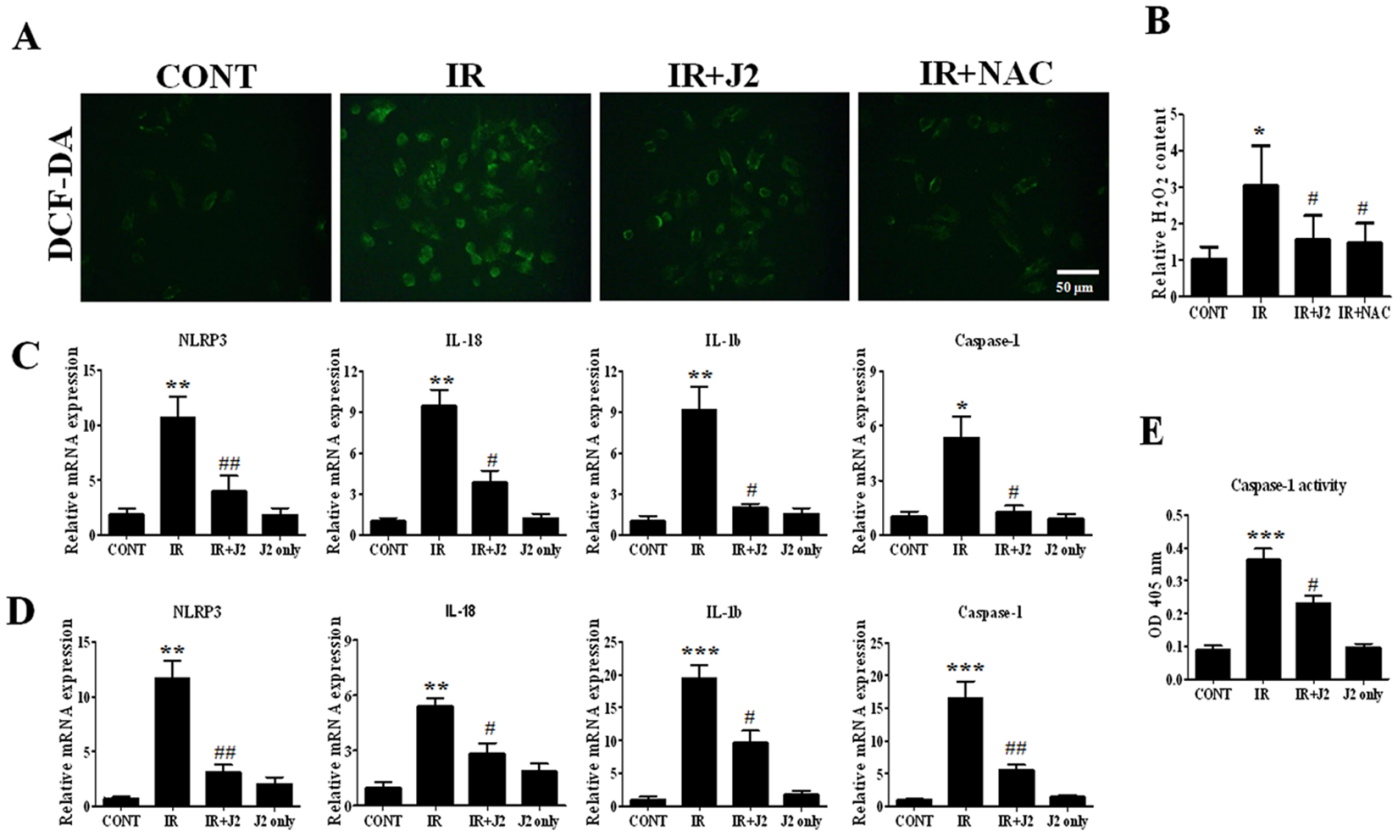
Effect of HSP27 inhibitor on ROS generation and inflammasome activation. (**A**) L132 cells were pretreated with J2 (2 uM) or NAC (10 mM) for 3 h and then exposed to X-rays at 10 Gy for 6 h. Cells were stained with DCF-DA and observed under fluorescent microscope. (**B**) Mice were sacrificed at 2 weeks after irradiation, mice plasma were centrifuge at 10,000 rpm for 5 min to remove insoluble particles. The content of H_2_O_2_ within the plasma was measured at absorbance at 560 nm. Quantitative real-time PCR for inflammasome-related genes. cDNA was synthesized from the total RNAs extracted from irradiated L132 cells (**C**) and mouse lungs (**D**) exposed to X-rays and subjected to RT-PCR analysis. (**E**) L132 cells were pretreated with or without J2 (2 uM) and then exposed to x-rays at 10 Gy for 24 h. Caspase-1 activity from cell lysates was measured at absorbance at 405 nm.

Radiation damage is essentially caused by ROS; therefore, ROS scavenging is a powerful way to protect against radiation damage. Due to antioxidant property of J2, we hypothesized that J2 may have ROS scavenging activity on radioprotective function. To test this out, we treated cells with X-ray, and ROS formation was determined using 2ʹ,7ʹ-dichlorofluorescein diacetate (DCF-DA), an indicator of intracellular ROS formation. *N*-acetylcysteine (NAC) was used as a positive antioxidant control. As shown in Fig. 6A, ROS level was markedly increased in L-132 cells treated with X-ray, whereas J2-treated cells suppressed intracellular ROS production. J2 also decreased the accumulation of H_2_O_2_ in mice plasma by inducing radiation (Fig. 6B). NLRP3 inflammasome is known to play an important role in pathogenesis of radiation-induced lung inflammation, and ROS has been proposed as regulatory factors of NLRP3 inflammasome^23,24^. As J2 can attenuate IR-induced inflammation in lungs of mice, we further examined whether J2 showed anti-inflammatory effect by inhibiting activation of inflammasome. We found that mRNA expressions of NLRP3, caspase-1, IL-18, and IL-1b were markedly elevated in IR group compared to control group *in vivo* and *in vitro* (Fig. 6 C,D). Also, compared to IR group, these targets were significantly inhibited by J2. Activation of caspase-1, which is a key molecule of inflammasome, induces cleavage of pro-IL-1b and IL-18, enabling its biologically active form^25^. Therefore, we investigated the effect of J2 on radiation-induced caspase-1 activation. L-132 cells were irradiated to X-ray at a dose of 10 Gy for 24 h, with or without J2 treatment. We found that activation of caspase-1 by irradiation was blocked in J2 treatment cells (Fig. 6E). Therefore, our results indicated that J2 plays an important role in inflammasome activation that leads to radiation-induced inflammation.

### Overexpression of HSP27 exacerbates radiation-induced lung inflammation

**Figure 7:**
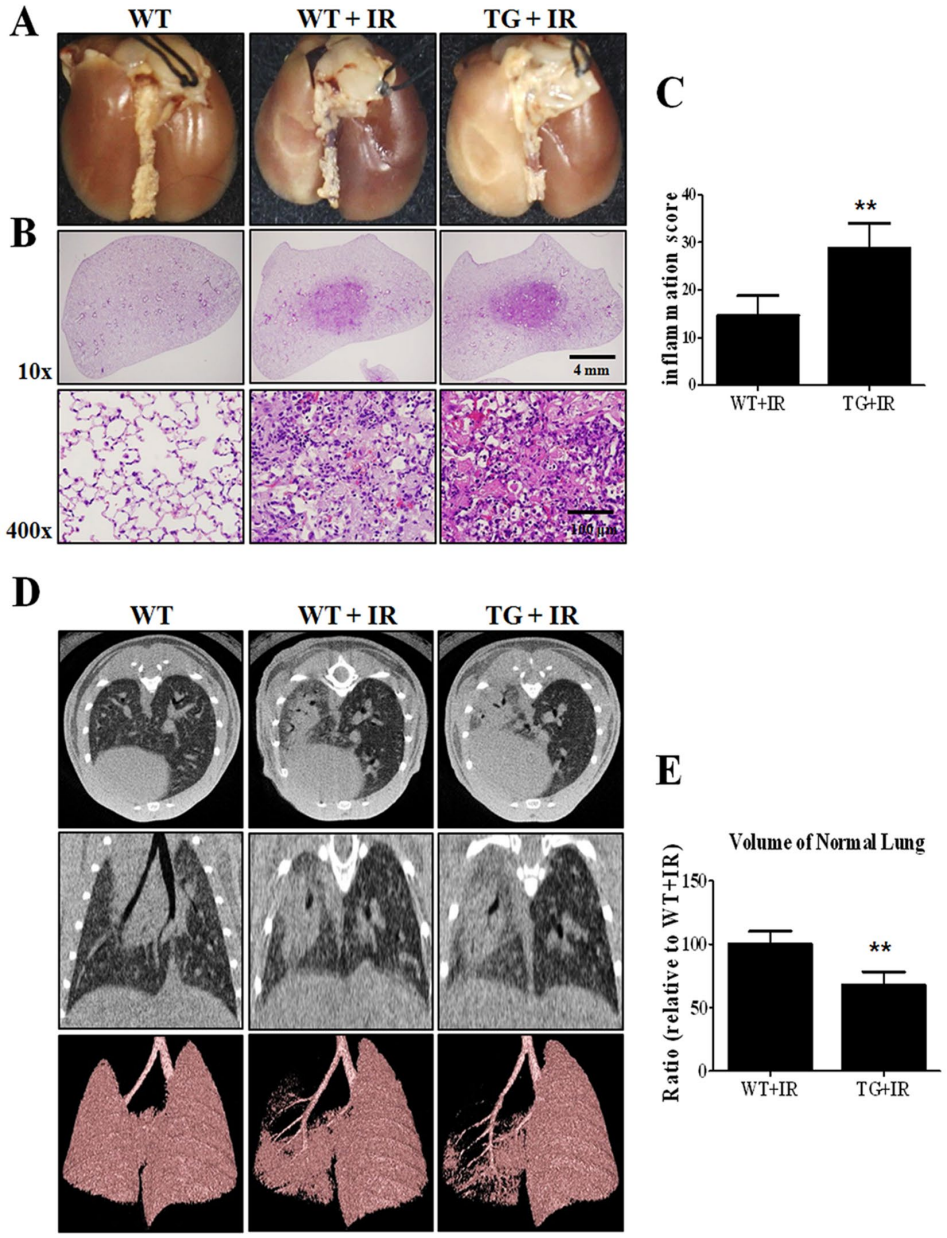
Gross morphological, histopathological and micro-CT analysis in HSP27 transgenic mice. (**A**) Representative gross finding. Mice were sacrificed at two weeks after irradiation. Four-percent paraformaldehyde was instilled via trachea, and lungs were immersed in fixation solution and photographed after complete fixation. (**B**) Haematoxylin and eosin-stained lung sections. (**C**) Graphs show quantification of inflammation score. (**D**) Representative micro-CT images of lungs of irradiated and control mice. Horizontal (top row), trans-axial (middle row), and 3D micro-CT (bottom row) images acquired at two weeks after irradiation. (E) Quantification of volume of normal left lung was presented as mean ± standard error. **P < 0.01 versus BL6 IR.

To determine whether HSP27 regulates radiation-induced lung inflammation, we observed the morphological, histological, and structural changes in HSP27 transgenic mice lungs. As shown in Fig. 7, radiation-induced lung inflammation significantly increased in TG + IR group compared to that in wild type irradiation group (WT + IR). Micro-CT images showed that normal lung volume in TG + IR group was significantly lower compared to that in WT + IR group, suggesting that HSP27 exacerbates radiation-induced lung inflammation.

## Discussion

Radiotherapy is an effective treatment for lung cancer. However, radiation-induced pneumonitis may develop, which can result in morbidity and mortality with severe side effects^26,27^. Therefore, the search for regulatory molecules in inflammatory response may be a new therapeutic strategy for radiation-induced lung injury.

In the current study, HSP27 expression significantly increased in lungs of irradiated mice (Supplementary Fig. S4), which were thought to be involved in radiation-induced lung injury.

Previously, we reported that altered dimerization of HSP27 inhibits its oligomerization and results in sensitization of tumors after combined treatment of conventional radiation and chemotherapy^10,11^. The J2, a synthetic chromone compound, promoted crosslinking into altered dimers, inhibiting HSP27 oligomerization without cytotoxicity^13^. These suggested that altered crosslinking is a powerful strategy for inhibition of HSP27 activity. In sh-HSP27 cells, there was no crosslinking of HSP27 protein by J2, indicating that crosslinking of HPS27 by J2 is specific for HSP27 protein^13^. In our study, we confirmed that J2 crosslinked HSP27 in a dose-dependent manner (Supplementary Fig. S1B). The current study also found, for the first time, that HSP27 inhibitor, J2, has a protective effect on radiation-induced lung inflammation, by using an ablative radiation-induced acute lung injury mouse model. In particular, the effect of radiation protection of J2 was investigated in comparison to amifostine, which was the first drug to be approved by the U.S. Food and Drug Administration as a radioprotective drug^18^, and the evidence of comparative evaluation of radiation protection effect of J2 was presented in our study.

To find the optimal dosage of J2 on X-ray-induced mouse lung inflammation model, we performed preliminary experiment using 7.5 and 15 mg/kg doses of J2. Our results showed that the dose of 15 mg/kg of J2 is likely enough concentration for eliciting inhibitory effects on radiation-induced lung inflammation. To find the optimal numbers of administration of J2 on X-ray-induced inflammation, we also performed preliminary experiment using single, once a week (two times for two weeks), and continual (six times for two weeks) treatment of J2. As a result, single and once a week treatment did not show significant effects on radiation-induced inflammation. Therefore, we decided to use continual treatment of J2 for the entire time duration after irradiation.

In order to investigate lung injury by irradiation, we first investigated gross and IHC staining. As shown in Fig. 1, irradiation resulted in severe lung injury in gross study, while a large amount of inflammatory cells infiltrated to irradiated injury sites in IHC study. Compared to IR group, treatment of J2 (IR + J2 group) reduced immune cell infiltration in IR + AMI group, and this reduction was also more effective. Real-time PCR results indicated that, while irradiation significantly increased expressions of cytokines (TGF-β, IL-6, IL-1b and IL-33) and chemokines (MIP1-α and CCL4), J2 treatment decreased expressions for most of these genes (Fig. 4). Such results suggest that J2 has anti-inflammatory activity against radiation injury.

Oxidative stress is involved in radiation-induced lung tissue damage. Actually, a number of basic studies have reported that a few antioxidants are effective in reducing oxidative damage; however, the same antioxidants failed to induce significant antioxidant effects in clinical studies^28^. Therefore, it is urgent to find a useful antioxidant to treat diseases that are caused by oxidative stress. In this study, we found that J2, an HSP27 inhibitor, suppressed ROS generation and oxidative stress by X-ray irradiation. However, other studies have shown that HSP27 suppressed increase of ROS^29^. There was also a discrepancy between our result and others’ findings. Therefore, we compared intracellular ROS generation by radiation between J2-treated cells and sh-HSP27 cells. Interestingly, in contrast to J2 treatment, sh-HSP27 cells showed increased production of ROS (Supplementary Fig. S5), suggesting that J2 itself, as a synthetic chromone compound (Supplementary Fig. S1A), might have ROS scavenging activity. Chromone (4H-chromen-4-ones) constitute an important class of oxygen-containing heterocyclic compound acknowledged by their antioxidant properties^30^. Recently, it has been reported that chromone structure-based compounds showed strong total antioxidant activities through radical scavenging and metal chelating^31^, which could support J2 have remarkable antioxidant activity. Therefore, we tested whether J2 has an antioxidant activity by using DPPH radical scavenging assay. As shown in Supplementary Fig. S3, J2 exhibited antioxidant activity in relatively dose-dependent manner, suggesting that J2 may have radioprotective activity on radiation injury. We showed that J2 and amifostine decreased radiation-induced oxidative stress by measuring 8-OHdG and NOX4 levels. However, J2 inhibited oxidative stress to a greater extent than amifostine (Fig. 5), suggesting that J2 is a stronger antioxidant than amifostine in the radiation-induced lung inflammation model. In a histological study, J2 reduced inflammation more effectively than amifostine (Fig. 1). Inflammatory response and oxidative stress play key roles in respiratory distress development^32,33^. In the present study, evaluation of the flexivent system showed that amifostine was less effective than J2 in respiratory distress recovery (Fig. 3). This is probably because J2 had more potent antioxidant activity than amifostine in the radiation-induced lung inflammation model used in this study, suggesting that J2 affected respiratory distress by regulating ROS production, oxidative stress, and lung inflammation.

Although a definitive mechanism of radiation-induced lung inflammation remains unknown, we previously reported that inflammasome is involved in radiation-induced lung inflammation^34^. Inflammasome activation triggers cleavage and activation of caspase-1, leading to immune response by promoting maturation of pro-inflammatory cytokines such as pro-IL-1b and pro-IL-18^25^. Therefore, regulation of inflammasome activation may reduce radiation-induced inflammation. In consistence with our previous report, expressions of inflammasome-related genes, *Nlrp3*, *Il18*, *Il-1b*, and *Casp1*, increased in irradiated lung tissues and L-132 cells. Such increase was reduced by treatment using J2 (Fig. 6), suggesting that J2 can suppress radiation-induced inflammasome activation pathways. Interleukin (IL)-1b is known to be a critical component of inflammasome^35^. Caspase-1 is activated by active inflammasomes, which cleave inactive forms of IL-1b into active forms^36^. In airway epithelial cells, increase of active IL-1b leads to release of pro-inflammatory cytokines, which plays an important role in inflammation of lung^37^. In the current study, IL-1b expression and caspase-1 activity dramatically increased during lung inflammation, indicating the presence of inflammasome activation; on the other hand, they were decreased by J2 treatment (Figs 4 and 6). While possible signalling pathways are needed to be further elucidated, activation of inflammasome signalling pathway plays an important role in the pathogenesis of radiation-induced pulmonary inflammation. Here, we present the possibility that J2 can attenuate IR-induced lung inflammation by suppressing activation of inflammasome signalling pathway *in vivo* and *in vitro*.

Micro-CT is used for quantitative analysis of early structural and histopathological changes associated with lung injury, and FlexiVent^TM^ is an achievable measurement system that directly evaluates lung function based on the same functional parameters as those used in humans^38^. Results of micro-CT analysis in the present study were in correlation with the histopathologic findings. Characteristic CT features of SBRT-induced lung injuries included ground-glass opacity and consolidation, which were observed at two weeks after irradiation in mice in our study. J2 treatment appears to have resulted in partial resolution of these features (Fig. 2). Results of FlexiVent^TM^ showed that J2-treated mice exhibited significantly better values of lung function parameters such as IC, Cst, and G than irradiated mice, suggesting that J2 treatment improves lung function. Particularly, our results showed that such protective effect of J2 against radiation-induced lung injury was more effective than that of amifostine. Taken together, our results suggest that J2 reduces radiation-induced lung inflammation, and therefore, may provide a good treatment method to overcome lung inflammation after radiation.

## Methods

### Animal experiment

All protocols involving the use of mice were approved by Animal Care and Use Committees of Yonsei University Medical School (2015–0267), and were performed in strict accordance to relevant guidelines. Male C57BL/6 mice (age, 6 weeks; weight, 20–25 g) were purchased from Charles River Korea (Orient Bio, Seongnam, South Korea), and were acclimatized (n = 5 per cage) for a week before irradiation. A single dose of 75 Gy was delivered to the left lung in a single fraction using image-guided small-animal irradiator (X-RAD 320; Precision, North Branford, CT, USA) that was equipped with a collimator system composed of 3.5-cm-thick copper to produce focal radiation beams, as well as an imaging subsystem consisting of a fluorescent screen coupled to a charge-coupled-device camera. We selected 3-mm collimators to mimic clinical SBRT conditions by irradiating only a small volume of tissue. The mice were divided into six groups (n = 6–8 per group) as follows: (1) control (C); (2) irradiation (IR) - mice were exposed to a single dose of 75 Gy delivered to the left lung in a single fraction; (3) irradiation + J2 (IR + J2) −15 mg/kg of J2 was intraperitoneal administered on every other day after irradiation; (4) irradiation + amifostine (IR + Ami) −100 mg/kg of amifostine were intraperitoneal administered on every other day after irradiation; (5) J2 only (J2) −15 mg/kg of J2 were intraperitoneal administered on every other day without irradiation; (6) Amifostine only (Ami) −100 mg/kg of amifostine were intraperitoneal administered on every other day without irradiation. On day 14, mice were sacrificed by CO_2_ asphyxiation, and their lung tissues were collected for analysis.

### Cell culture

L-132 human lung epithelial cells were grown in Dulbecco’s modified Eagle’s medium supplemented with 10% fetal bovine serum at 37 °C in a humidified 5% (v/v) CO_2_ atmosphere. Cells were seeded at 1.0 × 10^6^ cells/60 mm plate. After 24 h, cells were washed with serum-free medium, and stored prior to the experiments.

### Suppression of HSP27 expression by shRNA

Lentiviruses were used to create stable L-132 cell lines expressing shRNA for HSP27 with a puromycin-resistance gene. HSP27 shRNA plasmid (sc-2935-SH) and shRNA Plasmid Transfection Reagent (sc-108061) were from Santa Cruz Biotechnology. To generate sh-control and sh-HSP27 cells, cell lines were transduced with 1 mol of lentivirus, and selected by puromycin (1 g/mL) for at least one week.

### Preparation of J2

Compounds were synthesized as described in previous study^12^.

### Preparation of lung tissues for histology and immunohistochemistry

Left-lung tissues of irradiated mice were collected and fixed in 4% paraformaldehyde, and then embedded in paraffin. For histological study, 4-μm tissue sections were stained with haematoxylin and eosin (H & E) and immunohistochemical (IHC) stains. Morphological changes were observed under light microscope.

### Micro-computed tomographic analysis

Micro-computed tomography (CT) images were acquired by a volumetric CT scanner (NFRPolaris- G90MVC: NanoFocusRay, Iksan, South Korea) at 50 kVp, 180 µA, and 150 mGy (number of views, 700; frame rate, 142 ms). Images were reconstructed (image size, 1232 × 1120 pixels; number of slices, 512) by volumetric cone-beam reconstruction (Feldkamp-Davis-Kress method) using in-line/off-line modes. Volumetric analysis was performed using Image J software. In order to minimize inter-specimen variations in measurement, same level settings were used for analysis of all images.

### Functional assessment of lungs

Lung function in irradiated mice was evaluated by Flexivent system (Flexivent®; SCIREQ©, Montreal, QC, Canada), which measures flow-volume relationships in the respiratory system, including forced oscillation, to discriminate between airway and lung tissue variables^17^. Evaluations were performed according to manufacturer’s instructions. Briefly, after anesthetization, mice were connected to computer-controlled small-animal ventilator and quasi-sinusoidally ventilated with tidal volume of 10 mL/kg at frequency of 150 breaths per minute. Measurement commenced when a stable ventilation pattern, without obvious spontaneous ventilator effort, was observed at ventilation pressure tracing. All perturbations were performed sequentially until three acceptable measurements (coefficient of determination >0.95) were recorded for each subject, from which an average was calculated.

### Real-time reverse transcription-polymerase chain reaction and western blotting

RNA was isolated from lung tissues of mice by RNeasy Mini Kit (Qiagen, CA, USA), according to manufacturer’s instructions. Real-time reverse transcription-polymerase chain reaction (RT-PCR) was performed using Light Cycler 480 SYBR Green I master mix and Light Cycler 480 real-time PCR machine (Roche Applied Science, Indianapolis, IN, USA). Quantification was performed by comparative CT method (ΔΔCT). Data were obtained from three independent PCR experiments, and are represented as mean ± standard error (SE).

Specific mouse primer sequences used for amplification are listed in Supplementary Table S1. After extracted proteins were separated by sodium dodecyl sulfate polyacrylamide gel electrophoresis (SDS-PAGE), membranes were probed with primary antibody, followed by incubation with horseradish peroxidase-coupled secondary antibody. Detection was performed by chemiluminescence-based detection kit (Bio-Rad, Hercules, CA, USA).

### Hydrogen peroxidase assay

Mouse plasma was centrifuge at 10,000 rpm for 5 min to remove insoluble particles, and then the content of H_2_O_2_ was analyzed with hydrogen peroxide assay kit (Cell Biolab, San Diego, CA, USA) according to manufacturer’s instructions. Colorimetric probe reacted with H_2_O_2_ and horseradish peroxidase enzyme to produce a pink color, which was measured at absorbance at 560 nm. H_2_O_2_ content within samples was calculated by comparing the standard concentration curves.

### DPPH assay for radical scavenging activity

Microplate 2, 2-diphenyl-1-picrylhydrazyl (DPPH) assay was performed^39^. Briefly, in a 96-well plate, successive sample dilutions (standard stocks of different samples 5 mM) in triplicate received DPPH solution (40 μM in methanol) in a total volume of 0.2 mL, and absorbance was measured at 550 nm using a microplate reader.

### Caspase-1 activity assay

For caspase-1 activity assay, caspase-1 activity was measured using caspase-1/ICE colorimetric assay kit (Biovision Inc, #K111) according to manufacturer’s protocol.

### Generateion of HSP25 transgenic mouse

HSP25 mice were generated by Macrogen Inc., and mice were interbred and maintained in pathogen-free condition at Macrogen Inc. (Seoul, Korea). All manipulations were conducted under the approval of Macrogen’s Institutional Animal Care and Use Committee. PMSG and hCG were briefly treated into C57BL/6 N female mice for superovulation. PMSG (7.5IU) and hCG were IP injected at interval of 48 hours (5IU) to female mice of 5–8 weeks. After hCG injection, these female mice were mated with C57BL/6 N stud male mice. Next day, virginal plug was used to check sacrificed female mice and harvest fertilized embryo. Hsp25 DNA was co-microinjected into one cell embryos. Standard microinjection procedures were used for transgenic mice production (Macrogen, Seoul, Korea). Microinjection of 4ng/ul DNA was directly applied into male pronucleus of zygote using micromanipulator, and microinjected embryos were incubated at 37 °C for 1–2 hrs. Fourteen to 16 embryos injected at one-cell stage were transplanted into oviducts of pseudopregnant recipient mice (ICR) by surgical methods. After F0 were born, genotyping test using tail cut samples for the presence of transgene were performed, and PCR analysis was performed to confirm their genomic DNA. PCR screening was done by phenol-extraction method.

### Statistical analysis

Statistical analysis was performed using Prism 5 software (Graph Pad Software Inc., San Diego, CA, USA). Comparison of variables between control and radiation-treatment groups was performed by Mann-Whitney U test. P-values < 0.05 were considered statistically significant.

## Electronic supplementary material

Supplementary Information
