# Supplementary material for: HSP27 inhibitor attenuates radiation-induced pulmonary inflammation
Source: Sci Rep. 2018 Mar 8;8:4189. doi: 10.1038/s41598-018-22635-9 (PMC5843649; doi:10.1038/s41598-018-22635-9)
Supplement: Supplementary file 1 — Supplementary Information [file 41598_2018_22635_MOESM1_ESM.pdf]

Supplementary Information

Title : HSP27 inhibitor attenuates radiation-induced pulmonary inflammation

Jee-Youn Kim[1], Yong-Min An[1], Byeong Rok Yoo[1], Jin-Mo Kim[1], Song Yee Han[1], Younghwa Na[2*], Yun-Sil Lee[3*], Jaeho Cho[1*]

Supplementary Table S1.

The parameter description using the flexiVent™ system measurements

Parameter	Abbreviations	Descritption
Inspiratory Capacity	IC	IC is the volume difference between functional residual capacity (FRC) and total lung capacity (TLC), Also equaling tidal volume plus the inspiratory reserve volume
Quasi-static Compliance	Cst	The parameter K of the Salazar-Knowles equation reflects the curvature of the upper portion
Tissue Damping	G	Tissue damping is closely related to tissue resistance and reflects the energy dissipation in the lung tissues.
Tissue Elastance	H	The parameter H is closely related to tissue elastance and reflects the energy conservation in the lung tissues.
Newtonian Resistance	Rn	The Newtonian Resistance parameter of the Constant Phase Model represents the resistance of the central airway.
Airway constriction	Rrs	Resistance of the respiratory system

Supplementary Table S2.

The sequence of the primers used for real-time PCR

**Genes**	**Forward (5'-3')**	**Reverse (5'-3')**
TGF-β	AGCGGACTACTATGCTAAAG	CCAAGGTAACGCCAGGAATT
IL-6	CCGGAGAGGAGACTTCACA	TCCACGATTTCCCAGAGAAC
IL-1b	GCCCATCCTCTGTGACTCAT	AGGCCACAGGTATTTTGTCG
IL-33	ATTCCCCGGCAAAGTTCAG	AACGGAGTCTCATGCAGTAG
MIP-1a	ATGAAGGTCTCCACCACTGC	GATGAATTGGCGTGGAATCT
CCL-4	CCCACTTCCTGCTGTTTCTC	GTCTGCCTCTTTTGGTCAGG
NLRP3	TGCTCTTCACTGCTATCAAG	ACAAGCCTTTGCTCCAGACCC
IL-18	GTGAACCCCAGACCAGACTG	CCTGGAACACGTTTCTGAAA
Caspase-1	TCCGCGGTTGAATCCTTTTCA	ACCACAATTGCTGTGTGTGCG

Supplementary Figure S1

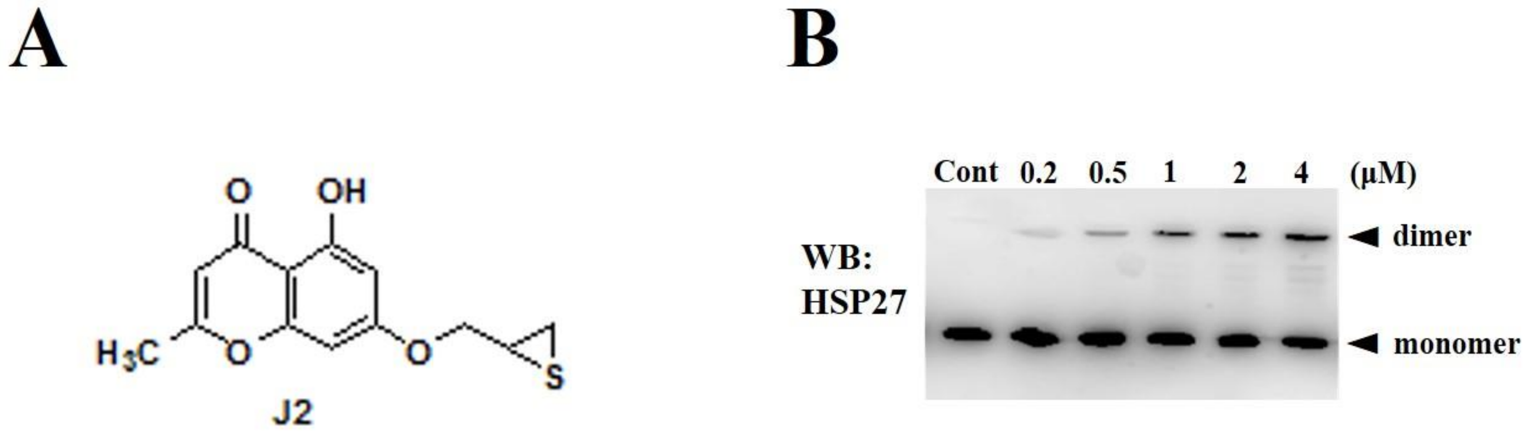

Fig. S1. J2 structure and HSP27 cross-linking activities of J2. (A) J2 structure. (B) HSP27 cross-linking activities of J2.

J2 was treated at each concentration for 24h in L132 cells. Western blots shows the expression of monomer and dimer form of HSP27.

Supplementary Figure S2

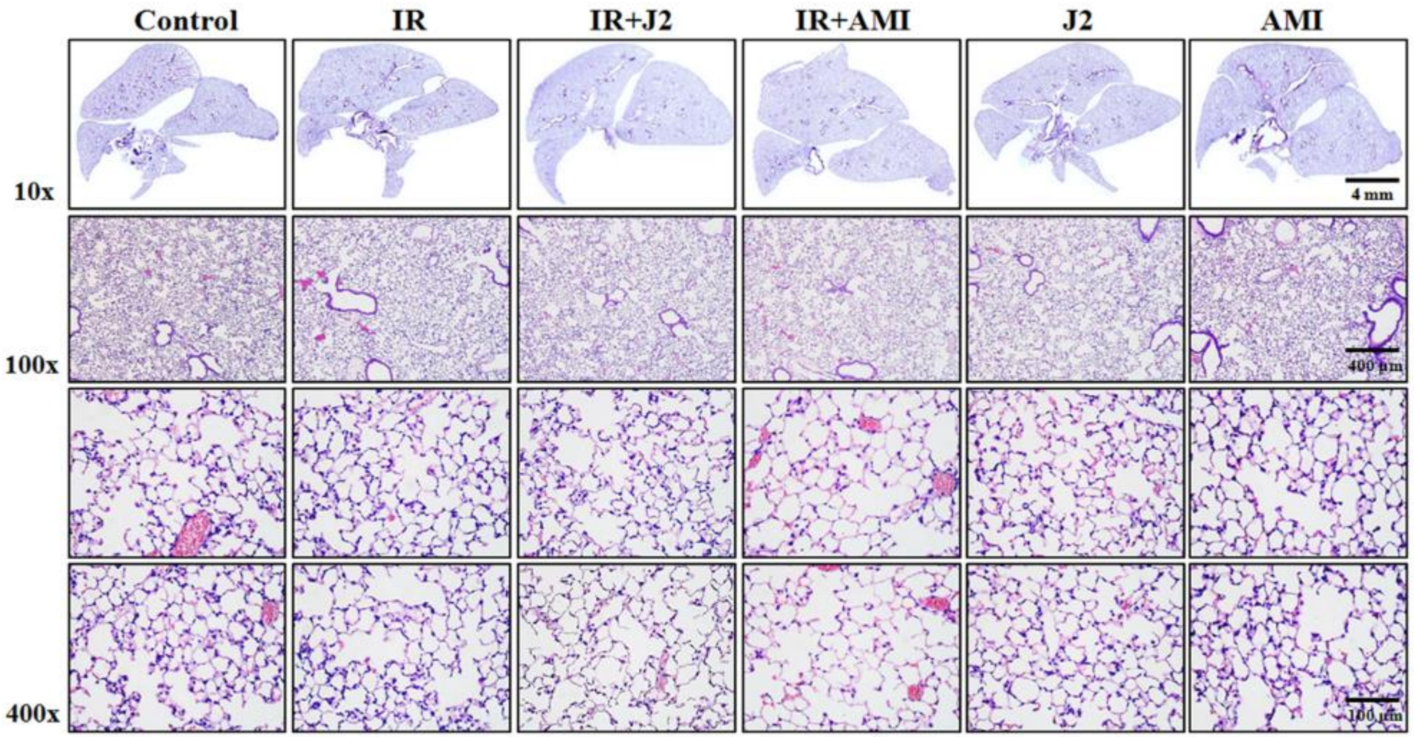

Fig. S2. Histological analysis in the right lung sections from the left lung-irradiated mice. Mice were sacrificed at 2 weeks after irradiation. Lung tissues were fixed in 4% paraformaldehyde and then dehydrated and embedded in paraffin. For histological study, 4 μm tissue sections were stained with haematoxylin and eosin (H & E), Magnification, 10x, 100x and 400x.

Supplementary Figure S3

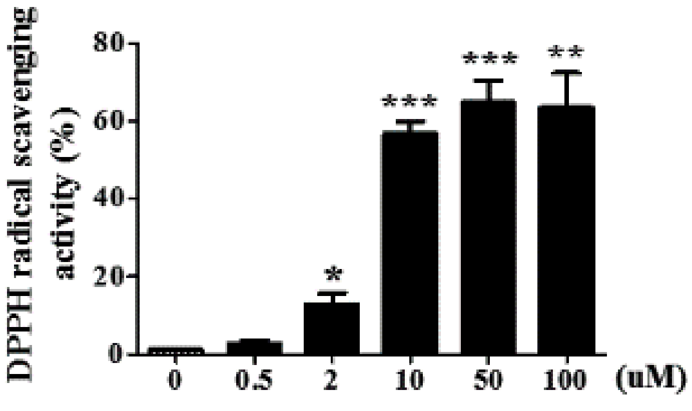

Fig. S3. Effect of J2 on DPPH radical scavenging activity. J2 exhibited radical scavenging activity in relatively dose-dependent manner. The values are expressed as mean ± SD of data from three independent assays (*$P < 0.05$, **$P < 0.01$ and ***$P < 0.001$ versus 0).

Supplementary Figure S4

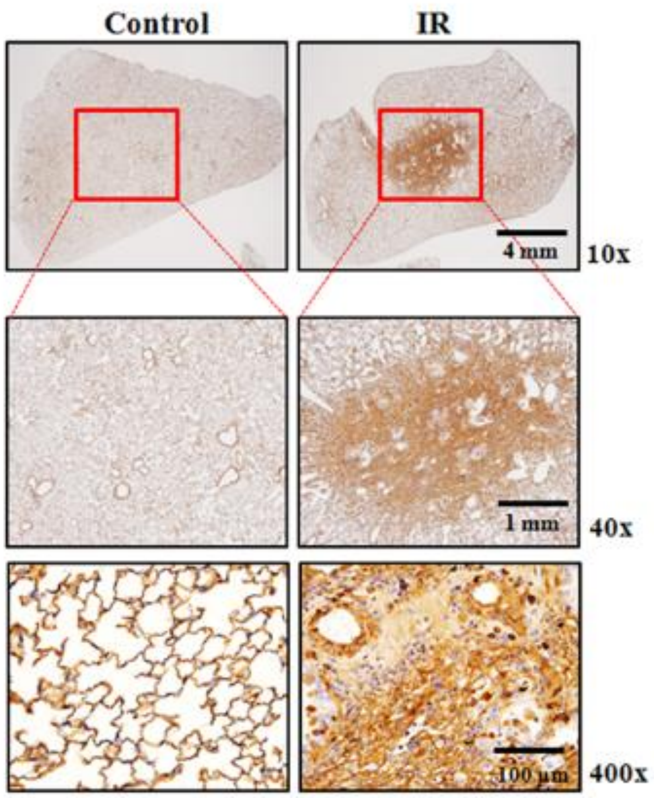

Fig. S4. HSP27 expression level after irradiation. Immunohistochemisty of HSP27 was performed in mouse lung tissues after focal 75 Gy irradiation (IR). HSP27 expression was dramatically increased by irradiation.

Supplementary Figure S5

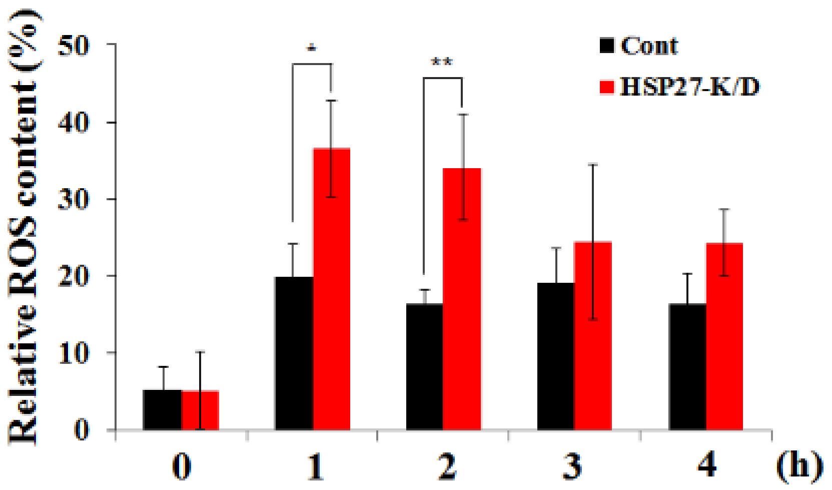

Fig. S5. Intracellular ROS generation by radiation between J2-treated cells and sh-HSP27 cells. Sh-control and sh-HSP27 cells treated with X-rays at 10 Gy for 0, 1, 2, 3 or 4 h, Cells were stained with DCF-DA and subjected to flow cytometric analysis.
